# DNA Damage Response and Repair in Adaptive Immunity

**DOI:** 10.3389/fcell.2022.884873

**Published:** 2022-05-17

**Authors:** Sha Luo, Ruolin Qiao, Xuefei Zhang

**Affiliations:** ^1^ Biomedical Pioneering Innovation Center, Innovation Center for Genomics, Peking University, Beijing, China; ^2^ Academy for Advanced Interdisciplinery Studies, Peking University, Beijing, China

**Keywords:** antibody diversification, RAG-initiated V(D)J recombination, AID-initiated CSR and SHM, DNA damage repair, cohesin-mediated loop extrusion

## Abstract

The diversification of B-cell receptor (BCR), as well as its secreted product, antibody, is a hallmark of adaptive immunity, which has more specific roles in fighting against pathogens. The antibody diversification is from recombination-activating gene (RAG)-initiated V(D)J recombination, activation-induced cytidine deaminase (AID)-initiated class switch recombination (CSR), and V(D)J exon somatic hypermutation (SHM). The proper repair of RAG- and AID-initiated DNA lesions and double-strand breaks (DSBs) is required for promoting antibody diversification, suppressing genomic instability, and oncogenic translocations. DNA damage response (DDR) factors and DSB end-joining factors are recruited to the RAG- and AID-initiated DNA lesions and DSBs to coordinately resolve them for generating productive recombination products during antibody diversification. Recently, cohesin-mediated loop extrusion is proposed to be the underlying mechanism of V(D)J recombination and CSR, which plays essential roles in promoting the orientation-biased deletional end-joining . Here, we will discuss the mechanism of DNA damage repair in antibody diversification.

## Introduction

**FIGURE 1:**
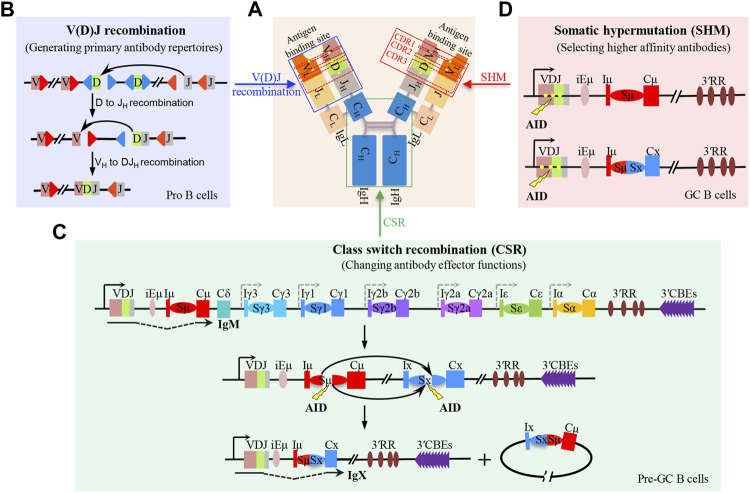
V(D)J recombination, class switch recombination, and V(D)J exon somatic hypermutation-mediated antibody diversification. **(A)** Schematic structure of antibody which is composed of two pairs of immunoglobulin heavy (IgH) and light (IgL) chains. The blue box indicates the antibody variable region which binds to antigens. The green box indicates the antibody constant region, where the class switch recombination occurs. The red box indicates the mutated region including CDR1, CDR2, and CDR3 within the V(D)J exon; the yellow dots indicate the mutation sites. **(B)** Two-step process of RAG-initiated V(D)J recombination in progenitor (pro) B cells. **(C)** Process of AID-initiated class switch recombination (CSR) in mature B cells before entering the germinal center (GC), termed as pre-GC cells. **(D)** Process of AID-initiated V(D)J exon somatic hypermutation (SHM) in non-switched and switched GC B cells.

The B-cell receptor (BCR) and antibody comprise two pairs of immunoglobulin heavy (IgH) and light (IgL) chains (Hwang et al., 2015). The N-terminal regions of IgH and IgL are the variable regions, which form the antigen-binding domain of BCR. The C-terminal region of IgH is the constant region that specifies the antibody effector function (Figure 1A) (Alt et al., 2013). In developing B cells, V(D)J recombination generates highly diverse antigen receptor repertoires by assembling the numerous IgH germline V_H_ (variable), D (diversity), and J_H_ (joining) gene segments in different combinations (Figure 1B). Also, IgL variable region exons are subsequently assembled by joining V_L_ and J_L_ segments (Ebert et al., 2015; Outters et al., 2015). In a given developing B cell, the unique IgH and IgL chains generate sets of mature B cells that express a highly diverse repertoire of BCR. In peripheral lymphoid organs, mature B cells can be activated by encountering antigens to undergo IgH class switch recombination (CSR) (Figure 1C) and V(D)J exon somatic hypermutation (SHM) (Figure 1D) to further diversify BCR/antibody affinity and function, enhancing antigen elimination (Methot and Di Noia, 2017; Yeap and Meng, 2019).

**FIGURE 2:**
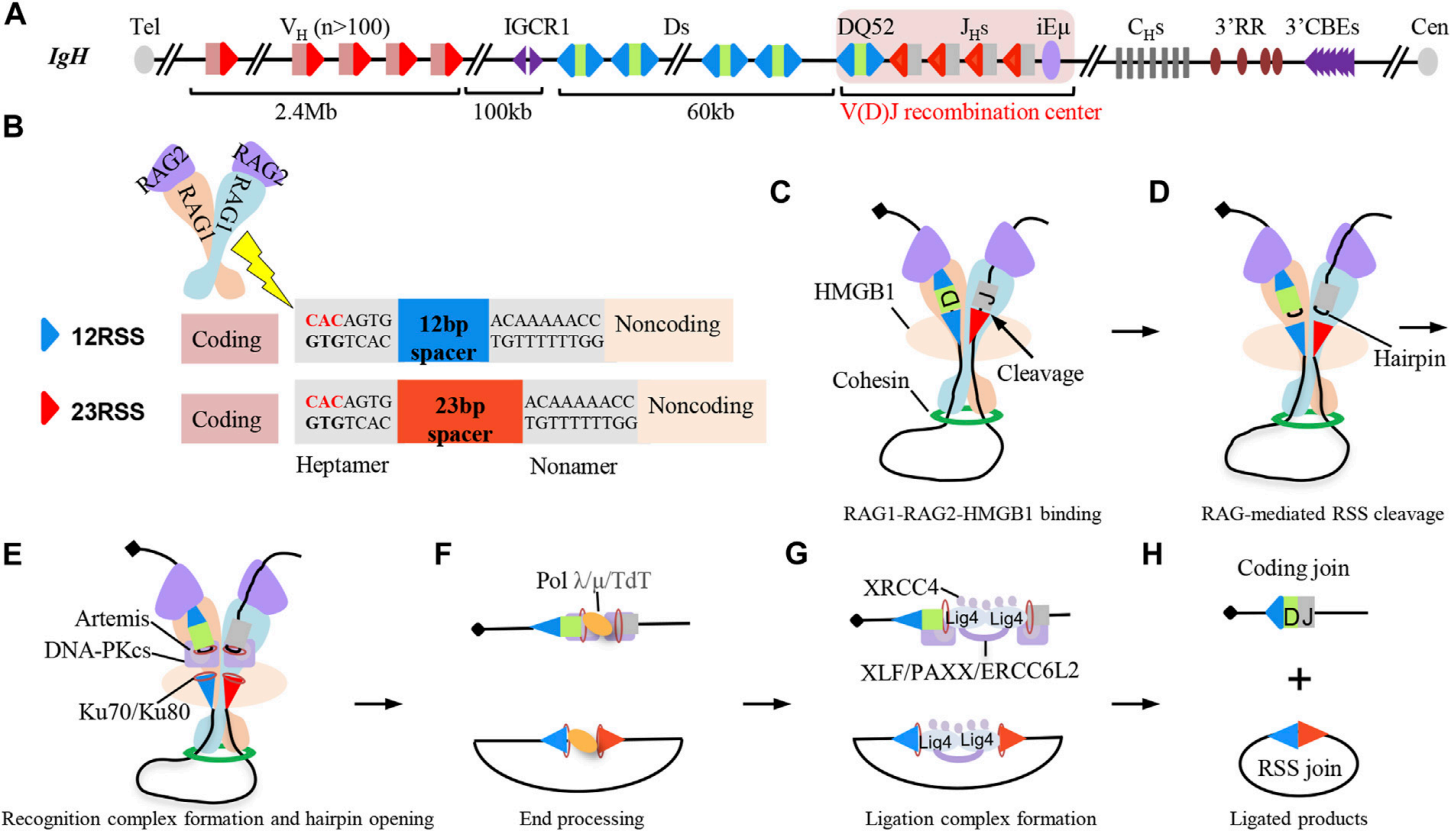
Classical non-homologous end-joining (C-NHEJ) joins RAG-initiated recombination signal sequence (RSS) breaks to complete V(D)J recombination. **(A)** Schematic structure of the IgH locus in mice. There are over one hundred V_H_ segments, 9–12 D segments, and 4 J_H_ segments in mouse IgH locus. Each V_H_ is downstream flanked by 23RSS. Each D is flanked by 12RSS on both sides. Each J_H_ is upstream flanked by 12RSS. **(B)** Schematic structure of RAG endonuclease, 12RSS (blue triangles), and 23RSS (red triangles). RAG cleaves the heptamer of RSS sequences. **(C–H)** C-NHEJ-mediated end-joining process during D to J_H_ recombination. RAG and HMGB1 bind to a pair of D and J_H_ segments for cleavage, and the synapsis of D and J_H_ segments is promoted by loop extrusion-mediated RAG scanning process **(C)**. RAG cuts the synapsed RSSs associated with the D and J_H_ segments to generate a pair of blunt RSS ends and a pair of hairpin-associated coding ends **(D)**. Ku70/Ku80 complex binds to the RAG-initiated DSBs and recruits DNA-PKcs-Artemis complex to open the coding end-associated hairpins **(E)**. RAG-initiated breaks can further be processed by DNA polymerase λ/μ and terminal deoxynucleotidyl transferase (TdT) **(F)**. XRCC4 and ligase 4 are recruited to the breaks to ligate the processed DNA breaks, and other redundant C-NHEJ factors including XLF, PAXX, and ERCC6L2 are also involved in the ligation step **(G)**.Final D to J_H_ recombination products include the coding join and RSS join **(H)**.

The mouse IgH locus spans 2.7 Mb with more than 100 functional V_H_s in the 2.4 Mb distal region, a 100 Kb intervening region, and a 60 Kb region with multiple Ds followed by 4 J_H_s (Figure 2A) (Ebert et al., 2015). V(D)J recombination is initiated by the Y-shaped recombination-activating gene (RAG) endonuclease (Liu et al., 2021). RAG is recruited to the V(D)J recombination center (RC), which includes the J_H_-proximal DQ52, 4 J_H_s, and the intronic enhancer iEμ (Teng and Schatz, 2015). RAG binds and cleaves the recombination signal sequences (RSSs) (Kim et al., 2015; Ru et al., 2015; Kim et al., 2018; Ru et al., 2018) that flank V_H_, D, and J_H_ gene segments (Figure 2B). The two blunt RSS ends are fused by classical non-homologous end-joining (C-NHEJ) directly to generate RSS joins as excision cycles, while the two coding ends are fused by C-NHEJ to generate the coding joins after DNA-PKcs and Artemis-mediated removal of coding end-associated hairpins (Figures 2C–H) (Zhao et al., 2020). V(D)J recombination is ordered, with Ds joining to J_H_s, prior to V_H_s joining to DJ_H_ intermediates to form V(D)J exons (Figure 1B) (Alt et al., 2013).

After V(D)J recombination is completed, immature B cells migrate to some peripheral lymphoid organs such as the spleen and further develop to become mature B cells (Nagasawa, 2006). Without stimulation or antigen activation, naïve B cells express the recombined V(D)J exon and its proximal Cμ exons that specify the IgM antibodies. Upon activation, mature B cells undergo CSR to replace the donor Cμ with one of the six sets of constant region exons (C_H_s) that lie 100–200 kb downstream, to change the antibody isotype with different pathogen-elimination functions (Figure 1C) (Yeap and Meng, 2019). Each C_H_ has an inducible (I) promoter exon, long (1–12 kb) repetitive switch (S) region, and several C_H_ exons (Hwang et al., 2015). Activation-induced cytidine deaminase (AID) (Muramatsu et al., 2000) initiates CSR by generating deamination lesions at frequent short DNA target motifs within donor Sμ, and a downstream acceptor S region (Hwang et al., 2015). The lesions are converted into DNA double-strand breaks (DSBs) by co-opting DNA damage repair factors. The upstream Sμ DSB ends are end-joined to the downstream acceptor S region DSB ends to complete CSR by C-NHEJ and alternative end-joining (A-EJ) (Boboila et al., 2012; Methot and Di Noia, 2017).

The switched and non-switched mature B cells can enter the lymphoid germinal centers (GCs), where they are further matured by introducing somatic hypermutation (SHM) into the V(D)J exons (Figure 1D) (Pilzecker and Jacobs, 2019; Roco et al., 2019). In response to antigen activation, AID targets the same deamination motifs in V(D)J exons that are mainly converted into mutational outcomes in GC B cells (Hwang et al., 2015). The mutated V(D)J exons that have higher binding affinity to the antigen are selected and expanded (Lau and Brink, 2020). This SHM process allows cellular selection to promote BCR/antibody affinity maturation.

## V(D)J Recombination

### RAG Initiates DNA Breaks for V(D)J Recombination

RAG endonuclease is a Y-shaped heterotetramer, which contains two units of RAG1 catalytic enzymes and two units of RAG2 regulatory co-factors (Kim et al., 2015; Ru et al., 2015; Kim et al., 2018; Ru et al., 2018). Both RAG1 and RAG2 are required for the physiological V(D)J recombination (Schatz et al., 1989; Oettinger et al., 1990). RAG1 has the DNA binding and cleaving activity to cut the heptamer of RSSs to generate blunt RSS ends and hairpin-associated coding ends (Figures 2B–D) (McBlane et al., 1995; van Gent et al., 1995; Alt et al., 2013). RAG1 interacts with numerous nucleolar proteins to modulate recombination activity in the nucleus (Brecht et al., 2020), and the N-terminal region of RAG1 regulates the efficiency and pathways of synapsis for V(D)J recombination (Beilinson et al., 2021). RAG2 has no DNA cleavage activity, but it is required to enhance RAG1 catalytic activity. RAG2 binds to DNA by recognizing trimethylation of lysine 4 on histone H3 (H3K4me3), which is a histone marker of active chromatin including promoters and enhancers (Matthews et al., 2007; Teng et al., 2015). The abundance of RAG2 protein is cell cycle-dependent which undergoes ubiquitin-dependent degradation when lymphocytes transit from G1 to the S phase (Li et al., 1996; Teng and Schatz, 2015). Also, RAG2 interacts with RAG1 to abolish RAG1 aggregation to initiate V(D)J recombination during the G1 phase (Brecht et al., 2020; Gan et al., 2021). The regulation of RAG2 promotes RAG-mediated V(D)J recombination in B cells during the G1 phase; meanwhile, it suppresses the generation of undesired DSBs and translocations to ensure the genome stability.

### Loop Extrusion-Mediated RAG Scanning Promotes V(D)J Recombination

RAG not only binds the bona fide RSSs flanked by the V, D, and J gene segments for physiological V(D)J recombination but also can capture and cut cryptic targets besides RSSs at a low frequency (Hu et al., 2015), which might lead to translocations related to B- and T-cell lymphoma (Mahowald et al., 2008). RAG can generate robust recombination between Dβ1 and Jβ1-1 when the Dβ1 and Jβ1-1 segments of T-cell receptor β (TCRβ) are inserted into the c-Myc locus (c-Myc-DJβ cassette). Meanwhile, the c-Myc-DJβ cassette insertion activates RAG activity to capture and cut the cryptic targets (convergent-orientated “CAC” motifs) linearly. Interestingly, RAG cryptic targets are restricted to the 1.8 Mb c-Myc domain anchored by CTCF binding elements (CBEs). Also, RAG cryptic activity within a domain also applies to other domains across the genome (Hu et al., 2015). Moreover, RAG extends its activity to the cryptic targets outside of a domain by deleting the CBE-mediated boundaries (Hu et al., 2015; Zhang Y. et al., 2019). This evidence suggests that RAG scans linearly to capture and cut the convergent-orientated CAC motifs within a domain.

The RAG scanning process can also explain the physiological D to J_H_ recombination and V_H_ to DJ_H_ recombination. The plasmid-based studies indicate that the RSS sequence, not RAG scanning, determines the utilization of D-RSSs (Gauss and Lieber, 1992), while the high-throughput HTGTS-V(D)J-seq analysis of large amounts of D-RSS-inverted *v-Abl* progenitor (pro)-B-cell lines supports that RSS orientation, not the RSS sequence, plays a key role in deletional D to J_H_ recombination, indicating that RAG scanning promotes the utilization of the downstream D-RSSs during physiological D to J_H_ recombination (Figure 2C) (Zhang Y. et al., 2019). J_H_-RSS-bound RAG initiates scanning from RC to the upstream D segments until aligning and cutting one downstream D-RSS with J_H_-RSS, leading to the generation of DJ_H_ recombination products (Figures 2C–H) (Zhang Y. et al., 2019). After DJ_H_ recombination, DJ_H_-RSS-bound RAG initiates scanning to the upstream V_H_ segments and cuts a convergent-orientated V_H_-RSS with DJ_H_-RSS to complete the V_H_ to DJ_H_ recombination, which is supported by the V_H_ inversion experiments in mice (Hill et al., 2020; Dai et al., 2021). The V_H_ region inversion eliminates V_H_ utilization and increases the utilization of newly formed CAC motifs within the inverted region, which strongly supports that RAG scanning promotes the capture of convergent-orientated V_H_-RSS in the physiological V_H_ to DJ_H_ recombination (Dai et al., 2021).

D to J_H_ joining occurs within the loop domain anchored upstream by the two divergent CBEs-formed IGCR1 between D and V_H_ and downstream by the ten tandem CBE-formed super anchor (3′CBEs) (Guo et al., 2011; Alt et al., 2013). V_H_ to DJ_H_ recombination needs the neutralization of IGCR1 anchor and V_H_-associated CBEs, which allows RAG scanning to the upstream V_H_s (Guo et al., 2011; Alt et al., 2013; Jain et al., 2018). The depletion of CTCF in *v-Abl* pro-B cells increases the utilization of distal V_H_s, indicating that RAG scans through the CBEs after removing CTCF-mediated anchors in *v-Abl* pro-B cells (Ba et al., 2020). Moreover, the depletion of Wapl, a cohesin unloader, in *v-Abl* pro-B cells also increases the utilization of distal V_H_s (Dai et al., 2021), which is consistent with the downregulation of Wapl in normal pro-B cells. It is likely that downregulated Wapl might neutralize CBE-mediated blocks to enhance RAG scanning to the upstream V_H_s, leading to the generation of more diverse antibody repertoires during physiological V(D)J recombination. RAG activity mainly focuses on the targets within the dynamic chromatin impediments including the CTCF-bound chromatin, highly transcribed chromatin, RAG-bound chromatin, and even catalytic-dead Cas9-bound chromatin (Zhang Y. et al., 2019; Zhang et al., 2022). The aforementioned evidence strongly supports that cohesin-mediated loop extrusion is the underlying mechanism of RAG scanning-mediated V(D)J recombination.

### DSB Response Factors Have Modest or No Effects on V(D)J Recombination

Intrinsic and extrinsic stress-induced DSBs are the most harmful DNA lesions to genome integrity, which trigger DNA damage response (DDR) by recruiting DDR factors to the DSBs for repairing. ATM and its downstream phosphorylated targets (H2AX, 53BP1, and MDC1) are the key DDR factors, which play crucial roles in repairing general DSBs and maintaining genome stability (Weitering et al., 2021).

RAG-initiated DSBs also recruit DDR factors during V(D)J recombination. ATM and ATM-phosphorylated p53 are recruited to the RAG-initiated DSBs to surveil the intermediates in V(D)J recombination, protecting against the potentially aberrant oncogenic translocations (Perkins et al., 2002). Also, coding joining is decreased with more un-joined coding ends in ATM-deficient pre-B cells, indicating that ATM stabilizes RAG-initiated DSBs during V(D)J recombination (Bredemeyer et al., 2006). 53BP1-deficient mice have relatively normal B-cell compartments and no substantial block in V(D)J recombination (Manis et al., 2004), while 53BP1-deficiency is also found to impair the distal V to DJ joining at the TCRα locus, suggesting a specific role of 53BP1 in maintaining genomic stability during long-range joining of DSBs (Difilippantonio et al., 2008). H2AX is recruited to the RAG-initiated DSBs at the TCRα locus (Chen et al., 2000), while it is not required for coding join formation or lymphocyte development (Bassing et al., 2002), suggesting that it only functions as a general surveillance machinery to prevent translocations during V(D)J recombination (Yin et al., 2009). MDC1-deficiency has no major block for V(D)J recombination or lymphocyte development (Lou et al., 2006). The recently identified shieldin complex, composed of MAD2L2/REV7, SHLD1, SHLD2, and SHLD3, is also dispensable for V(D)J recombination and lymphocyte development (Ghezraoui et al., 2018; Ling et al., 2020). Altogether, DDR factors have relatively modest or no effect on V(D)J recombination, suggesting the redundant roles of these DDR factors with others during V(D)J recombination (more discussion in the next section).

### C-NHEJ Exclusively Joins RAG-Initiated Breaks During V(D)J Recombination

Intrinsic and extrinsic stress-induced DSBs are mainly repaired by homologous recombination (HR) and C-NHEJ. HR mainly functions in the late S and G2 phases, which uses sister chromatids as templates for error-free DNA repair. C-NHEJ repairs almost all DSBs outside of S and G2 phases and is the major DSB repair pathway in both dividing and non-dividing cells (Zhao et al., 2020).

RAG-initiated DSBs are exclusively repaired by C-NHEJ, resulting from the synapsis of breaks held by the RAG post-cleavage complex (PCC) (Figure 2D) (Teng and Schatz, 2015; Libri et al., 2021). RAG2 truncations or charge-neutralizing mutations switch the DSB repair pathway from C-NHEJ to alternative end-joining (A-EJ) and HR (Corneo et al., 2007; Coussens et al., 2013; Gigi et al., 2014). RAG interacts with the core NHEJ factors Ku70/Ku80 (Figure 2E) (Raval et al., 2008), and Ku70 suppresses A-EJ in G1-arrested pro-B cells (Liang et al., 2021). The deficiency of Ku70 has a severe combined immunodeficiency (SCID) phenotype and severely impairs the formation of coding joins and RSS joins (Gu et al., 1997; Ouyang et al., 1997). The deficiency of Ku80 arrests lymphocyte development at early progenitor stages and induces a profound impairment in V(D)J recombination (Nussenzweig et al., 1996; Zhu et al., 1996). The Ku70/80 complex recruits another two core C-NHEJ factors, namely, XRCC4 and ligase 4, to the DSBs for end joining (Figure 2G). XRCC4 is a scaffolding protein to stabilize ligase 4 to form the ligation complex for ligating the DSB ends. XRCC4- or ligase 4-deficient mice die during the late embryonic development, resulting from the p53-dependent apoptosis (Barnes et al., 1998; Frank et al., 1998; Gao et al., 1998). Deleting p53 in XRCC4-deficient or ligase 4-deficient mice rescues the lethality, while has no rescues for the impaired V(D)J recombination and lymphocyte development (Frank et al., 1998; Gao et al., 2000). So the four core C-NHEJ factors are absolutely required for V(D)J recombination.

In addition to the conserved core C-NHEJ factors, there are several other C-NHEJ factors including DNA-PKcs, Artemis, XLF, and PAXX. DNA-PKcs is recruited to the RAG-initiated coding ends (Lieber, 2010) and phosphorylates Artemis to activate its endonuclease activity, leading to the removal of the coding end-associated hairpins (Figure 2E) (Ma et al., 2002). Before the DNA-PKcs-Artemis-processed coding ends get joined, DNA polymerases (Polμ, Polλ) and terminal deoxynucleotidyl transferase (TdT)-mediated nucleotide additions can further increase the junction diversity (Figure 2F) (Zhao et al., 2020). DNA-PKcs not only play roles in processing coding ends for coding joins, but also functions in RSS joins. The deficiency of DNA-PKcs and DDR factors severely impairs RSS joins, suggesting DNA-PKcs has redundant roles with DDR factors in RSS joins (Gapud et al., 2011; Zha et al., 2011b). In contrast to other C-NHEJ factors, XLF seems to be dispensable for V(D)J recombination as the deficiency of XLF has no measurable impact on V(D)J recombination (Li et al., 2008), while V(D)J recombination is almost abrogated by the deficiency of both XLF and ATM or one of its downstream DDR factors, suggesting functional redundancy of XLF with DDR factors during V(D)J recombination (Zha et al., 2011a; Liu et al., 2012; Oksenych et al., 2012). PAXX, a paralog of XLF, is also dispensable for V(D)J recombination, but the deficiency of both PAXX and XLF almost abrogates V(D)J recombination (Kumar et al., 2016). The new identified ERCC6L2 interacts with other C-NHEJ factors and plays functionally redundant roles with XLF during V(D)J recombination (Figure 2G) (Liu et al., 2020). These aforementioned C-NHEJ factors have relatively less influence on V(D)J recombination than the core C-NHEJ factor, resulting from the functional redundancy with DDR factors or other unknown factors.

## Class Switch Recombination and Somatic Hypermutation

### AID-Initiated DNA Lesions for CSR and V(D)J Exon SHM

**FIGURE 3:**
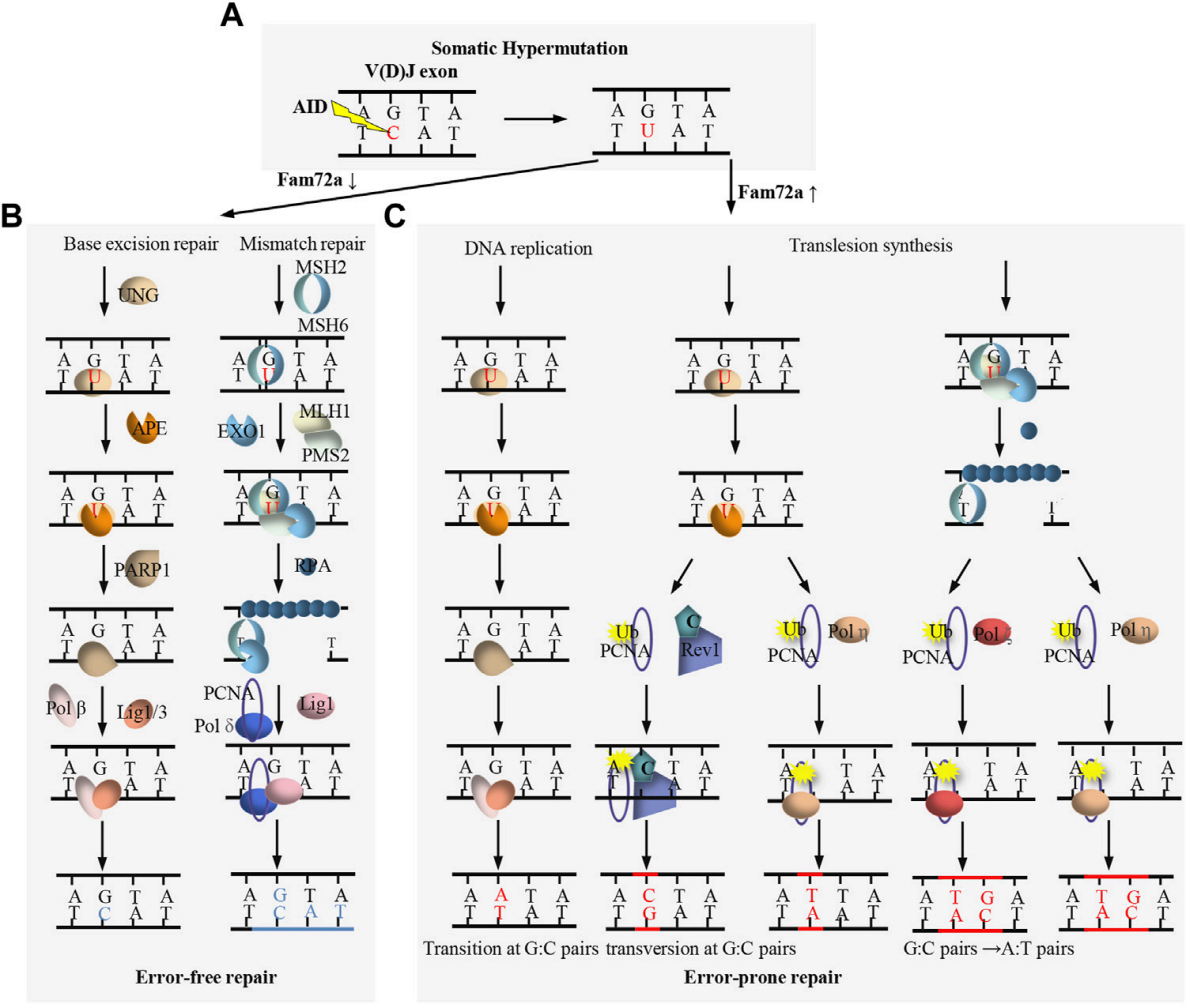
Overview of DNA damage repair process during SHM. **(A)** AID targets the dC to generate dU within V(D)J exon. **(B)** FAM72a downregulation promotes base excision repair (BER)- and mismatch repair (MMR)-mediated error-free DNA repair. **(C)** FAM72a upregulation promotes BER- and MMR-mediated error-prone DNA repair, leading to the mutation of V(D)J exon during SHM.

**FIGURE 4:**
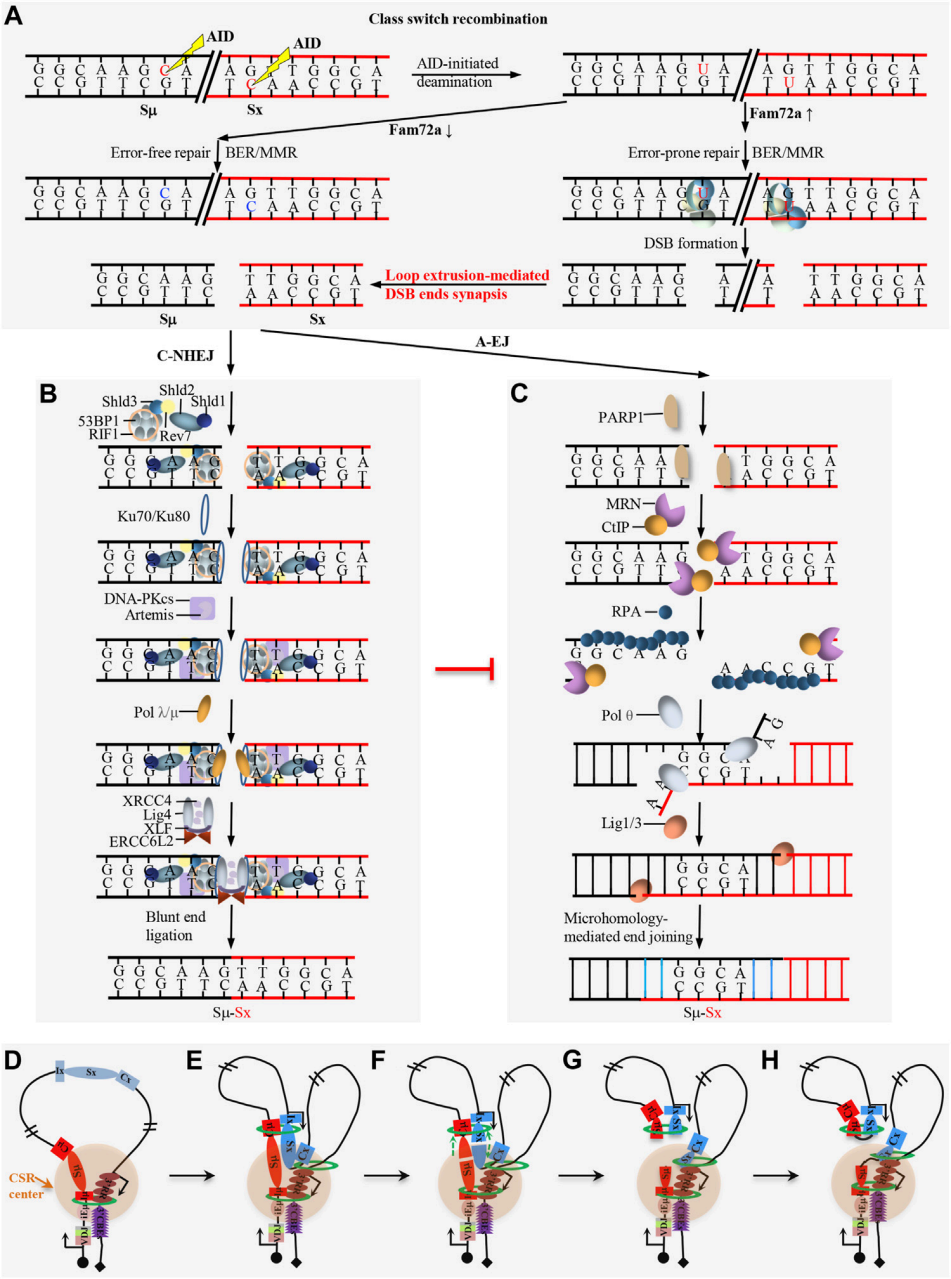
Overview of DNA damage repair process during CSR. **(A)** FAM72a regulates the error-prone vs. error-free DNA repair during AID-initiated CSR. AID-initiated breaks are converted into double-strand breaks (DSBs) upon high level of FAM72a during CSR. **(B)** Overview of the DNA damage response (DDR) factors and C-NHEJ in promoting direct end joining during CSR. **(C)** Overview of the alternative end joining (A-EJ) in promoting DSB end resection and microhomology-mediated end joining during CSR. **(C–H)** Loop extrusion-mediated CSR model. Loop extrusion promotes CSR center formation **(D)**, acceptor S region activation **(E)**, Sμ–Sx synapsis **(F)**, and deletional end joining **(G–H)**.

AID is essential for both CSR and SHM (Muramatsu et al., 2000). As a paralog of the RNA-cytosine deaminase APOBEC family, AID is originally proposed to be an RNA editing enzyme (Muramatsu et al., 1999; Muramatsu et al., 2000), while large amount of evidence supports that AID functions as a DNA deaminase to deaminate deoxycytidine (dC) to deoxyuridine (dU) (Feng et al., 2020). AID preferentially targets the dC in short DGYW (D = A/G/T, Y=C/T, W = A/T) motifs within the V(D)J exons (Figure 3A) and S regions (Figure 4A) for SHM and CSR, respectively (Rogozin and Diaz, 2004). AID-initiated dU causes the mismatch with deoxyguanine (dG), which can be converted into the point mutation or DSB by base excision repair (BER) and mismatch repair (MMR) during SHM and CSR (Figures 3C, 4A) (Hwang et al., 2015; Methot and Di Noia, 2017).

BER and MMR are two complex DNA repair processes which can function as error-free repair and error-prone repair (Figures 3B,C) (Hwang et al., 2015; Methot and Di Noia, 2017). BER repairs the AID-initiated dU from the recognition and excision of dU by UNG. APE cleaves the DNA to generate a nick at the UNG-initiated abasic site. The nick is further processed to generate a gap, which is filled by DNA polymerase β and sealed by DNA ligase 1/3. MMR repairs the AID-initiated dU from the recognition of the mismatch by MSH2/6, which further recruits MLH1 and PMS2. Exo1 excises the DNA sequences adjacent to the mismatch to generate a gap, which is filled by DNA polymerase δ and sealed by DNA ligase 1. Instead of accurate repair by BER and MMR (Figure 3B), mutagenic repair frequently occurs after AID-initiated dU during CSR and SHM (Figures 3C, 4A). Recent studies indicate that FAM72a influences the usage of error-prone vs. error-free DNA repair by regulating UNG2 abundance during CSR and SHM (Figures 3B,C, 4A) (Feng et al., 2021; Rogier et al., 2021).

### DDR Factors Play Essential Roles for AID-Initiated CSR

DDR factors can also be recruited to the AID-initiated DNA lesions, and these DDR factors are required for CSR as the deficiency of the individual ATM, H2AX, or 53BP1 decreases the CSR frequency (Reina-San-Martin et al., 2003; Lumsden et al., 2004; Manis et al., 2004; Reina-San-Martin et al., 2004; Franco et al., 2006; Reina-San-Martin et al., 2007; Bothmer et al., 2010). 53BP1 and H2AX are the downstream targets of ATM, but 53BP1-deficiency has a much more dramatic effect than that of ATM- or H2AX-deficiency (Dong et al., 2015; Panchakshari et al., 2018). RIF1 is a downstream factor of 53BP1 to inhibit DSB end resection and RIF1-deficiency significantly decreases CSR (Chapman et al., 2013; Di Virgilio et al., 2013). The shieldin complex functions downstream of 53BP1-RIF1 pathway and the deficiency of shieldin components have similar phenotype as that of 53BP1-deficiency (Xu et al., 2015; Dev et al., 2018; Ghezraoui et al., 2018; Gupta et al., 2018; Noordermeer et al., 2018). This 53BP1 pathway can compete with MRN/CtIP activity to protect DNA ends during CSR (Mirman and de Lange, 2020). The deficiency of these DDR factors variably increases the resection of AID-initiated DSBs and increases the utilization of longer microhomology for end joining (Dong et al., 2015; Panchakshari et al., 2018). This evidence indicates that DDR factors inhibit resection to maintain the integrity of AID-initiated DSBs for the efficient C-NHEJ pathway (Figure 4B), while the deficiency of DDR factors switches the end joining from C-NHEJ to the less efficient A-EJ which is prone to use longer microhomology (Figure 4C).

### End Joining of the AID-Initiated DSBs During CSR

The core C-NHEJ factor deficiency completely abolishes V(D)J recombination and blocks lymphocyte development, while core C-NHEJ factor-deficiency only decreases but not abrogates CSR, suggesting other less efficient end-joining pathways can join the AID-initiated breaks when C-NHEJ is absent during CSR (Yan et al., 2007; Boboila et al., 2012). This less efficient end-joining pathway is identified as A-EJ (Boboila et al., 2012) (Figure 4C).

C-NHEJ is the major DSB end-joining pathway during CSR (Figure 4B). The deficiency of the individual core C-NHEJ factor, Ku70, Ku80, XRCC4, or ligase 4, impairs CSR (Casellas et al., 1998; Manis et al., 1998; Pan-Hammarstrom et al., 2005; Yan et al., 2007; Han and Yu, 2008; Panchakshari et al., 2018). In addition to the core C-NHEJ factors, DNA-PKcs and Artemis are also necessary for joining AID-initiated DSBs during CSR (Franco et al., 2008). The deficiency of XLF impairs CSR (Zha et al., 2011a), while deficiency of PAXX, a paralog of XLF, has no influence on CSR (Kumar et al., 2016). ERCC6L2 is identified as a new NHEJ factor and ERCC6L2-deficiency impairs CSR. Surprisingly, ERCC6L2 deficiency does not increase the resection of AID-initiated break ends, but it significantly increases the inversional end joining during CSR (Liu et al., 2020). ERCC6L2 regulates the orientation-biased end joining without affecting the DSB end resection *via* an unprecedented mechanism during CSR.

A-EJ is activated when C-NHEJ or DDR factors are absent during CSR (Figure 4C). The deficiency of ligase 4 shares some similar features as that of DDR factor deficiency, including the increase of DSB resection, utilization of longer microhomology, and decrease of CSR frequency (Panchakshari et al., 2018). A-EJ is relatively less intelligible than C-NHEJ. PARP1 can respond to DNA damage and bind to the break sites during A-EJ (Wei and Yu, 2016). Then ligase 1 and ligase 3, the key joining factors of the A-EJ pathway, play redundant roles in joining AID-initiated DSBs during CSR (Lu et al., 2016; Masani et al., 2016). Several exonucleases and endonucleases can also enhance DSB resection and promote A-EJ during CSR (Bai et al., 2021; Sun et al., 2021). Further studies are required to figure out the whole picture of the A-EJ pathway in CSR and other physio-pathological processes.

### Loop Extrusion-Mediated CSR

AID-initiated CSR occurs within the ∼200 kb constant region of the IgH locus in mature B cells. Chromatin loop extrusion is proposed to be the underlying mechanism of CSR, which promotes the formation of the CSR center, transcriptional activation of acceptor S regions, synapsis of donor Sμ and an activated acceptor S region, and deletional joining of AID-initiated DSBs during CSR (Figure 4D) (Zhang X. et al., 2019; Zhang et al., 2021; Zhang et al., 2022). In addition to the physiological CSR process, the loop extrusion-mediated CSR model can also explain some abnormal switching events within the CSR center, including the IgH locus suicide recombination between Sμ and 3′RR (Peron et al., 2012), the ectopic S region switching after CBE insertion in the IgH constant region (Zhang X. et al., 2019) or 3′CBEs deletion (Zhang et al., 2021) and the Sμ-Sγ3 switching after inserting Sμ, Sγ3, and core 3′RR in the Igκ locus (Le Noir et al., 2021).

In resting B cells, cohesin is loaded onto either the active iEμ-Sμ region or the downstream 3′RR enhancer region to initiate loop extrusion. Cohesin-mediated loop extrusion brings these two active regions, namely, iEμ-Sμ and 3′RR into proximity to form a basal loop, in which the iEμ-Sμ and 3′RR serve as dynamic loop anchors. This basal loop is termed as a dynamic CSR center (Zhang X. et al., 2019). When B cells get activated, loop extrusion brings the primed acceptor S region into the CSR center, where it gets transcriptionally activated by 3′RR. Then, the activated acceptor S region loads cohesin to initiate loop extrusion to bring the donor Sμ into close proximity with the activated acceptor S region, leading to the synapsis of two S regions in the CSR center (Zhang X. et al., 2019).

AID can target different locations of the synapsed donor Sμ and acceptor S region at different times within the CSR center. Once AID initiates a DSB within an S region, the DSB ends will be pulled toward the opposite direction by loop extrusion and stalled by the associated cohesin rings. The two pairs of ends held by cohesin rings will be joined deletionally to generate the productive CSR products (Zhang X. et al., 2019). The disruption of the synapsis structure by inserting CBEs that have a convergent orientation to 3′CBEs between donor Sμ and acceptor Sα significantly increases the inversional joining without influencing DSB end resection, which means that the loop extrusion-mediated perfect synapsis of the donor Sμ and acceptor S region is required for the deletional end-joining during CSR (Zhang X. et al., 2019). Loop extrusion-mediated deletional end-joining is consistent with the cohesin accumulation at DSBs (Kim et al., 2002; Strom et al., 2004). Loop extrusion is also proposed to be the underlying mechanism of DNA damage repair. Loop extrusion-mediated ATM scanning along the chromatin adjacent to the DSB site phosphorylates H2AX until reaching the loop anchor to form DNA damage repair foci (Arnould et al., 2021), which shares some similar features to the loop extrusion-mediated deletional end-joining during CSR (Zhang X. et al., 2019). Loop extrusion might have more general roles in DNA damage repair, DSB end joining, and recombination processes.

### The Roles of DDR Factors in AID-Initiated SHM in GC B Cells

Upon activation by antigens, mature B cells can undergo CSR and SHM. CSR occurs prior to the mature B cells entering GC, where the V(D)J exons get mutated (Roco et al., 2019). Unlike the critical roles of DDR factors in CSR, ATM, 53BP1, and H2AX are dispensable for the V(D)J exon SHM. The deficiency of the individual ATM, 53BP1, or H2AX has no effect on the SHM frequency of the V(D)J exon (Reina-San-Martin et al., 2003; Manis et al., 2004; Reina-San-Martin et al., 2004). On the other hand, the checkpoint signaling *via* the ATR/Chk1 axis is downregulated by the transcription factor Bcl-6 in GC B cells, suggesting that negative regulation of the ATR/Chk1 axis is required for efficient SHM *in vivo* (Ranuncolo et al., 2007; Polo et al., 2008; Frankenberger et al., 2014; Bello and Jungnickel, 2021). However, Chk2 has opposite effects to Chk1 in the regulation of SHM. The deficiency of Chk2 decreases the SHM frequency, resulting from the defects of C-NHEJ and increase of the Chk1 activity (Davari et al., 2014). So, the ATR/Chk1/Chk2-mediated checkpoint signaling of the DNA damage response is crucial for the physiological SHM.

## Conclusion

BCRs and antibodies play vital roles in protecting against antigens. The diversification of BCRs and antibodies from RAG-initiated V(D)J recombination, AID-initiated CSR, and V(D)J exon SHM is crucial for efficient elimination of antigens. However, the mechanisms of these complicated antibody diversification processes are still not well understood. The immunoglobulin genes must be tightly regulated to generate the large amounts of highly efficient antibodies, meanwhile, suppress the generation of undesired translocations or mutations. So, there are still many puzzling questions: how do B cells minimize the off-target effects of RAG and AID during antibody diversification and what are the mechanisms of their specificities? How DNA repair factors/pathways are differentially regulated for the general DNA damage and immunoglobulin gene recombination? Whether cohesin-mediated loop extrusion plays more roles in antibody diversification? Answers to these questions provide not only insights into the understanding of antibody diversification during B-cell development but also the basis for understanding the immune-related diseases. Moreover, the mechanism of antibody diversification has a wide range of applications for drug development of related diseases such as COVID-19 and HIV.

## References

[B1] Alt F. W. Zhang Y. Meng F.-L. Guo C. Schwer B. (2013). Mechanisms of Programmed DNA Lesions and Genomic Instability in the Immune System. Cell 152 (3), 417–429. 10.1016/j.cell.2013.01.007 23374339 PMC4382911

[B2] Arnould C. Rocher V. Finoux A.-L. Clouaire T. Li K. Zhou F. (2021). Loop Extrusion as a Mechanism for Formation of DNA Damage Repair Foci. Nature 590 (7847), 660–665. 10.1038/s41586-021-03193-z 33597753 PMC7116834

[B3] Ba Z. Lou J. Ye A. Y. Dai H.-Q. Dring E. W. Lin S. G. (2020). CTCF Orchestrates Long-Range Cohesin-Driven V(D)J Recombinational Scanning. Nature 586 (7828), 305–310. 10.1038/s41586-020-2578-0 32717742 PMC7554077

[B4] Bai W. Zhu G. Xu J. Chen P. Meng F. Xue H. (2021). The 3′-flap Endonuclease XPF-ERCC1 Promotes Alternative End Joining and Chromosomal Translocation during B Cell Class Switching. Cel Rep. 36 (13), 109756. 10.1016/j.celrep.2021.109756 34592150

[B5] Barnes D. E. Stamp G. Rosewell I. Denzel A. Lindahl T. (1998). Targeted Disruption of the Gene Encoding DNA Ligase IV Leads to Lethality in Embryonic Mice. Curr. Biol. 8 (25), 1395–1398. 10.1016/s0960-9822(98)00021-9 9889105

[B6] Bassing C. H. Chua K. F. Sekiguchi J. Suh H. Whitlow S. R. Fleming J. C. (2002). Increased Ionizing Radiation Sensitivity and Genomic Instability in the Absence of Histone H2AX. Proc. Natl. Acad. Sci. U.S.A. 99 (12), 8173–8178. 10.1073/pnas.122228699 12034884 PMC123040

[B7] Beilinson H. A. Glynn R. A. Yadavalli A. D. Xiao J. Corbett E. Saribasak H. (2021). The RAG1 N-Terminal Region Regulates the Efficiency and Pathways of Synapsis for V(D)J Recombination. J. Exp. Med. 218 (10), e20210250. 10.1084/jem.20210250 34402853 PMC8374863

[B8] Bello A. Jungnickel B. (2021). Impact of Chk1 Dosage on Somatic Hypermutation *In Vivo* . Immunol. Cel Biol 99 (8), 879–893. 10.1111/imcb.12480 34042197

[B9] Boboila C. Alt F. W. Schwer B. (2012). Classical and Alternative End-Joining Pathways for Repair of Lymphocyte-specific and General DNA Double-Strand Breaks. Adv. Immunol. 116, 1–49. 10.1016/B978-0-12-394300-2.00001-6 23063072

[B10] Bothmer A. Robbiani D. F. Feldhahn N. Gazumyan A. Nussenzweig A. Nussenzweig M. C. (2010). 53BP1 Regulates DNA Resection and the Choice between Classical and Alternative End Joining during Class Switch Recombination. J. Exp. Med. 207 (4), 855–865. 10.1084/jem.20100244 20368578 PMC2856023

[B11] Brecht R. M. Liu C. C. Beilinson H. A. Khitun A. Slavoff S. A. Schatz D. G. (2020). Nucleolar Localization of RAG1 Modulates V(D)J Recombination Activity. Proc. Natl. Acad. Sci. U.S.A. 117 (8), 4300–4309. 10.1073/pnas.1920021117 32047031 PMC7049140

[B12] Bredemeyer A. L. Sharma G. G. Huang C.-Y. Helmink B. A. Walker L. M. Khor K. C. (2006). ATM Stabilizes DNA Double-Strand-Break Complexes during V(D)J Recombination. Nature 442 (7101), 466–470. 10.1038/nature04866 16799570

[B13] Casellas R. Nussenzweig A. Wuerffel R. Pelanda R. Reichlin A. Suh H. (1998). Ku80 Is Required for Immunoglobulin Isotype Switching. EMBO J. 17 (8), 2404–2411. 10.1093/emboj/17.8.2404 9545251 PMC1170583

[B14] Chapman J. R. Barral P. Vannier J.-B. Borel V. Steger M. Tomas-Loba A. (2013). RIF1 Is Essential for 53BP1-dependent Nonhomologous End Joining and Suppression of DNA Double-Strand Break Resection. Mol. Cel 49 (5), 858–871. 10.1016/j.molcel.2013.01.002 PMC3594748 23333305

[B15] Chen H. T. Bhandoola A. Difilippantonio M. J. Zhu J. Brown M. J. Tai X. (2000). Response to RAG-Mediated VDJ Cleavage by NBS1 and Gamma-H2ax. Science 290 (5498), 1962–1965. 10.1126/science.290.5498.1962 11110662 PMC4721589

[B16] Corneo B. Wendland R. L. Deriano L. Cui X. Klein I. A. Wong S. Y. (2007). Rag Mutations Reveal Robust Alternative End Joining. Nature 449 (7161), 483–486. 10.1038/nature06168 17898768

[B17] Coussens M. A. Wendland R. L. Deriano L. Lindsay C. R. Arnal S. M. Roth D. B. (2013). RAG2's Acidic Hinge Restricts Repair-Pathway Choice and Promotes Genomic Stability. Cell Rep 4 (5), 870–878. 10.1016/j.celrep.2013.07.041 23994475 PMC4008148

[B18] Dai H. Q. Hu H. Lou J. Ye A. Y. Ba Z. Zhang X. (2021). Loop Extrusion Mediates Physiological Igh Locus Contraction for RAG Scanning. Nature 590 (7845), 338–343. 10.1038/s41586-020-03121-7 33442057 PMC9037962

[B19] Davari K. Frankenberger S. Schmidt A. Tomi N. S. Jungnickel B. (2014). Checkpoint Kinase 2 Is Required for Efficient Immunoglobulin Diversification. Cell Cycle 13 (23), 3659–3669. 10.4161/15384101.2014.964112 25483076 PMC4614315

[B20] Dev H. Chiang T. W. Lescale C. de Krijger I. Martin A. G. Pilger D. (2018). Shieldin Complex Promotes DNA End-Joining and Counters Homologous Recombination in BRCA1-Null Cells. Nat. Cel Biol 20 (8), 954–965. 10.1038/s41556-018-0140-1 PMC6145444 30022119

[B21] Di Virgilio M. Callen E. Yamane A. Zhang W. Jankovic M. Gitlin A. D. (2013). Rif1 Prevents Resection of DNA Breaks and Promotes Immunoglobulin Class Switching. Science 339 (6120), 711–715. 10.1126/science.1230624 23306439 PMC3815530

[B22] Difilippantonio S. Gapud E. Wong N. Huang C. Y. Mahowald G. Chen H. T. (2008). 53BP1 Facilitates Long-Range DNA End-Joining during V(D)J Recombination. Nature 456 (7221), 529–533. 10.1038/nature07476 18931658 PMC3596817

[B23] Dong J. Panchakshari R. A. Zhang T. Zhang Y. Hu J. Volpi S. A. (2015). Orientation-specific Joining of AID-Initiated DNA Breaks Promotes Antibody Class Switching. Nature 525 (7567), 134–139. 10.1038/nature14970 26308889 PMC4592165

[B24] Ebert A. Hill L. Busslinger M. (2015). Spatial Regulation of V-(D)J Recombination at Antigen Receptor Loci. Adv. Immunol. 128, 93–121. 10.1016/bs.ai.2015.07.006 26477366

[B25] Feng Y. Li C. Stewart J. A. Barbulescu P. Seija Desivo N. Alvarez-Quilon A. (2021). FAM72A Antagonizes UNG2 to Promote Mutagenic Repair during Antibody Maturation. Nature 600 (7888), 324–328. 10.1038/s41586-021-04144-4 34819670 PMC9425297

[B26] Feng Y. Seija N. Di Noia J. M. Martin A. (2020). AID in Antibody Diversification: There and Back Again. Trends Immunol. 41 (7), 586–600. 10.1016/j.it.2020.04.009 32434680 PMC7183997

[B27] Franco S. Gostissa M. Zha S. Lombard D. B. Murphy M. M. Zarrin A. A. (2006). H2AX Prevents DNA Breaks from Progressing to Chromosome Breaks and Translocations. Mol. Cel 21 (2), 201–214. 10.1016/j.molcel.2006.01.005 16427010

[B28] Franco S. Murphy M. M. Li G. Borjeson T. Boboila C. Alt F. W. (2008). DNA-PKcs and Artemis Function in the End-Joining Phase of Immunoglobulin Heavy Chain Class Switch Recombination. J. Exp. Med. 205 (3), 557–564. 10.1084/jem.20080044 18316419 PMC2275379

[B29] Frank K. M. Sekiguchi J. M. Seidl K. J. Swat W. Rathbun G. A. Cheng H. L. (1998). Late Embryonic Lethality and Impaired V(D)J Recombination in Mice Lacking DNA Ligase IV. Nature 396 (6707), 173–177. 10.1038/24172 9823897

[B30] Frankenberger S. Davari K. Fischer-Burkart S. Bottcher K. Tomi N. S. Zimber-Strobl U. (2014). Checkpoint Kinase 1 Negatively Regulates Somatic Hypermutation. Nucleic Acids Res. 42 (6), 3666–3674. 10.1093/nar/gkt1378 24423870 PMC3973322

[B31] Gan T. Wang Y. Liu Y. Schatz D. G. Hu J. (2021). RAG2 Abolishes RAG1 Aggregation to Facilitate V(D)J Recombination. Cel Rep 37 (2), 109824. 10.1016/j.celrep.2021.109824 PMC8783374 34644584

[B32] Gao Y. Ferguson D. O. Xie W. Manis J. P. Sekiguchi J. Frank K. M. (2000). Interplay of P53 and DNA-Repair Protein XRCC4 in Tumorigenesis, Genomic Stability and Development. Nature 404 (6780), 897–900. 10.1038/35009138 10786799

[B33] Gao Y. Sun Y. Frank K. M. Dikkes P. Fujiwara Y. Seidl K. J. (1998). A Critical Role for DNA End-Joining Proteins in Both Lymphogenesis and Neurogenesis. Cell 95 (7), 891–902. 10.1016/s0092-8674(00)81714-6 9875844

[B34] Gapud E. J. Dorsett Y. Yin B. Callen E. Bredemeyer A. Mahowald G. K. (2011). Ataxia Telangiectasia Mutated (Atm) and DNA-PKcs Kinases Have Overlapping Activities during Chromosomal Signal Joint Formation. Proc. Natl. Acad. Sci. U S A. 108 (5), 2022–2027. 10.1073/pnas.1013295108 21245316 PMC3033293

[B35] Gauss G. H. Lieber M. R. (1992). The Basis for the Mechanistic Bias for Deletional over Inversional V(D)J Recombination. Genes Dev. 6 (8), 1553–1561. 10.1101/gad.6.8.1553 1644296

[B36] Ghezraoui H. Oliveira C. Becker J. R. Bilham K. Moralli D. Anzilotti C. (2018). 53BP1 Cooperation with the REV7-Shieldin Complex Underpins DNA Structure-specific NHEJ. Nature 560 (7716), 122–127. 10.1038/s41586-018-0362-1 30046110 PMC6989217

[B37] Gigi V. Lewis S. Shestova O. Mijuskovic M. Deriano L. Meng W. (2014). RAG2 Mutants Alter DSB Repair Pathway Choice *In Vivo* and Illuminate the Nature of 'alternative NHEJ. Nucleic Acids Res. 42 (10), 6352–6364. 10.1093/nar/gku295 24753404 PMC4041462

[B38] Gu Y. Seidl K. J. Rathbun G. A. Zhu C. Manis J. P. van der Stoep N. (1997). Growth Retardation and Leaky SCID Phenotype of Ku70-Deficient Mice. Immunity 7 (5), 653–665. 10.1016/s1074-7613(00)80386-6 9390689

[B39] Guo C. Yoon H. S. Franklin A. Jain S. Ebert A. Cheng H. L. (2011). CTCF-binding Elements Mediate Control of V(D)J Recombination. Nature 477 (7365), 424–430. 10.1038/nature10495 21909113 PMC3342812

[B40] Gupta R. Somyajit K. Narita T. Maskey E. Stanlie A. Kremer M. (2018). DNA Repair Network Analysis Reveals Shieldin as a Key Regulator of NHEJ and PARP Inhibitor Sensitivity. Cell 173 (4), 972–988 e923. 10.1016/j.cell.2018.03.050 29656893 PMC8108093

[B41] Han L. Yu K. (2008). Altered Kinetics of Nonhomologous End Joining and Class Switch Recombination in Ligase IV-Deficient B Cells. J. Exp. Med. 205 (12), 2745–2753. 10.1084/jem.20081623 19001141 PMC2585838

[B42] Hill L. Ebert A. Jaritz M. Wutz G. Nagasaka K. Tagoh H. (2020). Wapl Repression by Pax5 Promotes V Gene Recombination by Igh Loop Extrusion. Nature 584 (7819), 142–147. 10.1038/s41586-020-2454-y 32612238 PMC7116900

[B43] Hu J. Meyers R. M. Dong J. Panchakshari R. A. Alt F. W. Frock R. L. (2016). Detecting DNA Double-Stranded Breaks in Mammalian Genomes by Linear Amplification-Mediated High-Throughput Genome-wide Translocation Sequencing. Nat. Protoc. 11 (5), 853–871. 10.1038/nprot.2016.043 27031497 PMC4895203

[B44] Hu J. Zhang Y. Zhao L. Frock R. L. Du Z. Meyers R. M. (2015). Chromosomal Loop Domains Direct the Recombination of Antigen Receptor Genes. Cell 163 (4), 947–959. 10.1016/j.cell.2015.10.016 26593423 PMC4660266

[B45] Hwang J. K. Alt F. W. Yeap L. S. (2015). Related Mechanisms of Antibody Somatic Hypermutation and Class Switch Recombination. Microbiol. Spectr. 3 (1), 1. MDNA3-0037-2014. 10.1128/microbiolspec.MDNA3-0037-2014 PMC4481323 26104555

[B46] Jain S. Ba Z. Zhang Y. Dai H. Q. Alt F. W. (2018). CTCF-binding Elements Mediate Accessibility of RAG Substrates during Chromatin Scanning. Cell 174 (1), 102–116. e114. 10.1016/j.cell.2018.04.035 29804837 PMC6026039

[B47] Kim J. S. Krasieva T. B. LaMorte V. Taylor A. M. Yokomori K. (2002). Specific Recruitment of Human Cohesin to Laser-Induced DNA Damage. J. Biol. Chem. 277 (47), 45149–45153. 10.1074/jbc.M209123200 12228239

[B48] Kim M. S. Chuenchor W. Chen X. Cui Y. Zhang X. Zhou Z. H. (2018). Cracking the DNA Code for V(D)J Recombination. Mol. Cel 70 (2), 358–370. e354. 10.1016/j.molcel.2018.03.008 PMC5987255 29628308

[B49] Kim M. S. Lapkouski M. Yang W. Gellert M. (2015). Crystal Structure of the V(D)J Recombinase RAG1-RAG2. Nature 518 (7540), 507–511. 10.1038/nature14174 25707801 PMC4342785

[B50] Kumar V. Alt F. W. Frock R. L. (2016). PAXX and XLF DNA Repair Factors Are Functionally Redundant in Joining DNA Breaks in a G1-Arrested Progenitor B-Cell Line. Proc. Natl. Acad. Sci. U S A. 113 (38), 10619–10624. 10.1073/pnas.1611882113 27601633 PMC5035843

[B51] Lau A. W. Brink R. (2020). Selection in the Germinal center. Curr. Opin. Immunol. 63, 29–34. 10.1016/j.coi.2019.11.001 31835060

[B52] Le Noir S. Bonaud A. Herve B. Baylet A. Boyer F. Lecardeur S. (2021). IgH 3' Regulatory Region Increases Ectopic Class Switch Recombination. Plos Genet. 17 (2), e1009288. 10.1371/journal.pgen.1009288 33556079 PMC7869978

[B53] Li G. Alt F. W. Cheng H. L. Brush J. W. Goff P. H. Murphy M. M. (2008). Lymphocyte-specific Compensation for XLF/cernunnos End-Joining Functions in V(D)J Recombination. Mol. Cel 31 (5), 631–640. 10.1016/j.molcel.2008.07.017 PMC2630261 18775323

[B54] Li Z. Dordai D. I. Lee J. Desiderio S. (1996). A Conserved Degradation Signal Regulates RAG-2 Accumulation during Cell Division and Links V(D)J Recombination to the Cell Cycle. Immunity 5 (6), 575–589. 10.1016/s1074-7613(00)80272-1 8986717

[B55] Liang Z. Kumar V. Le Bouteiller M. Zurita J. Kenrick J. Lin S. G. (2021). Ku70 Suppresses Alternative End Joining in G1-Arrested Progenitor B Cells. Proc. Natl. Acad. Sci. U S A. 118 (21), e2103630118. 10.1073/pnas.2103630118 34006647 PMC8166026

[B56] Libri A. Marton T. Deriano L. (2021). The (Lack of) DNA Double-Strand Break Repair Pathway Choice during V(D)J Recombination. Front. Genet. 12, 823943. 10.3389/fgene.2021.823943 35082840 PMC8785701

[B57] Lieber M. R. (2010). The Mechanism of Double-Strand DNA Break Repair by the Nonhomologous DNA End-Joining Pathway. Annu. Rev. Biochem. 79, 181–211. 10.1146/annurev.biochem.052308.093131 20192759 PMC3079308

[B58] Ling A. K. Munro M. Chaudhary N. Li C. Berru M. Wu B. (2020). SHLD2 Promotes Class Switch Recombination by Preventing Inactivating Deletions within the Igh Locus. EMBO Rep. 21 (8), e49823. 10.15252/embr.201949823 32558186 PMC7403657

[B59] Liu C. Zhang Y. Liu C. C. Schatz D. G. (2021). Structural Insights into the Evolution of the RAG Recombinase. Nat. Rev. Immunol. 39 (21), e105857. 10.1038/s41577-021-00628-6 34675378

[B60] Liu X. Jiang W. Dubois R. L. Yamamoto K. Wolner Z. Zha S. (2012). Overlapping Functions between XLF Repair Protein and 53BP1 DNA Damage Response Factor in End Joining and Lymphocyte Development. Proc. Natl. Acad. Sci. U S A. 109 (10), 3903–3908. 10.1073/pnas.1120160109 22355127 PMC3309750

[B61] Liu X. Liu T. Shang Y. Dai P. Zhang W. Lee B. J. (2020). ERCC6L2 Promotes DNA Orientation-specific Recombination in Mammalian Cells. Cell Res 30 (9), 732–744. 10.1038/s41422-020-0328-3 32355287 PMC7608219

[B62] Lou Z. Minter-Dykhouse K. Franco S. Gostissa M. Rivera M. A. Celeste A. (2006). MDC1 Maintains Genomic Stability by Participating in the Amplification of ATM-dependent DNA Damage Signals. Mol. Cel 21 (2), 187–200. 10.1016/j.molcel.2005.11.025 16427009

[B63] Lu G. Duan J. Shu S. Wang X. Gao L. Guo J. (2016). Ligase I and Ligase III Mediate the DNA Double-Strand Break Ligation in Alternative End-Joining. Proc. Natl. Acad. Sci. U S A. 113 (5), 1256–1260. 10.1073/pnas.1521597113 26787905 PMC4747774

[B64] Lumsden J. M. McCarty T. Petiniot L. K. Shen R. Barlow C. Wynn T. A. (2004). Immunoglobulin Class Switch Recombination Is Impaired in Atm-Deficient Mice. J. Exp. Med. 200 (9), 1111–1121. 10.1084/jem.20041074 15504820 PMC2211853

[B65] Ma Y. Pannicke U. Schwarz K. Lieber M. R. (2002). Hairpin Opening and Overhang Processing by an Artemis/DNA-dependent Protein Kinase Complex in Nonhomologous End Joining and V(D)J Recombination. Cell 108 (6), 781–794. 10.1016/s0092-8674(02)00671-2 11955432

[B66] Mahowald G. K. Baron J. M. Sleckman B. P. (2008). Collateral Damage from Antigen Receptor Gene Diversification. Cell 135 (6), 1009–1012. 10.1016/j.cell.2008.11.024 19070571

[B67] Manis J. P. Gu Y. Lansford R. Sonoda E. Ferrini R. Davidson L. (1998). Ku70 Is Required for Late B Cell Development and Immunoglobulin Heavy Chain Class Switching. J. Exp. Med. 187 (12), 2081–2089. 10.1084/jem.187.12.2081 9625768 PMC2212369

[B68] Manis J. P. Morales J. C. Xia Z. Kutok J. L. Alt F. W. Carpenter P. B. (2004). 53BP1 Links DNA Damage-Response Pathways to Immunoglobulin Heavy Chain Class-Switch Recombination. Nat. Immunol. 5 (5), 481–487. 10.1038/ni1067 15077110

[B69] Masani S. Han L. Meek K. Yu K. (2016). Redundant Function of DNA Ligase 1 and 3 in Alternative End-Joining during Immunoglobulin Class Switch Recombination. Proc. Natl. Acad. Sci. U S A. 113 (5), 1261–1266. 10.1073/pnas.1521630113 26787901 PMC4747719

[B70] Matthews A. G. Kuo A. J. Ramon-Maiques S. Han S. Champagne K. S. Ivanov D. (2007). RAG2 PHD finger Couples Histone H3 Lysine 4 Trimethylation with V(D)J Recombination. Nature 450 (7172), 1106–1110. 10.1038/nature06431 18033247 PMC2988437

[B71] McBlane J. F. van Gent D. C. Ramsden D. A. Romeo C. Cuomo C. A. Gellert M. (1995). Cleavage at a V(D)J Recombination Signal Requires Only RAG1 and RAG2 Proteins and Occurs in Two Steps. Cell 83 (3), 387–395. 10.1016/0092-8674(95)90116-7 8521468

[B72] Methot S. P. Di Noia J. M. (2017). Molecular Mechanisms of Somatic Hypermutation and Class Switch Recombination. Adv. Immunol. 133, 37–87. 10.1016/bs.ai.2016.11.002 28215280

[B73] Mirman Z. de Lange T. (2020). 53BP1: a DSB Escort. Genes Dev. 34 (1-2), 7–23. 10.1101/gad.333237.119 31896689 PMC6938671

[B74] Muramatsu M. Kinoshita K. Fagarasan S. Yamada S. Shinkai Y. Honjo T. (2000). Class Switch Recombination and Hypermutation Require Activation-Induced Cytidine Deaminase (AID), a Potential RNA Editing Enzyme. Cell 102 (5), 553–563. 10.1016/s0092-8674(00)00078-7 11007474

[B75] Muramatsu M. Sankaranand V. S. Anant S. Sugai M. Kinoshita K. Davidson N. O. (1999). Specific Expression of Activation-Induced Cytidine Deaminase (AID), a Novel Member of the RNA-Editing Deaminase Family in Germinal center B Cells. J. Biol. Chem. 274 (26), 18470–18476. 10.1074/jbc.274.26.18470 10373455

[B76] Nagasawa T. (2006). Microenvironmental Niches in the Bone Marrow Required for B-Cell Development. Nat. Rev. Immunol. 6 (2), 107–116. 10.1038/nri1780 16491135

[B77] Noordermeer S. M. Adam S. Setiaputra D. Barazas M. Pettitt S. J. Ling A. K. (2018). The Shieldin Complex Mediates 53BP1-dependent DNA Repair. Nature 560 (7716), 117–121. 10.1038/s41586-018-0340-7 30022168 PMC6141009

[B78] Nussenzweig A. Chen C. da Costa Soares V. Sanchez M. Sokol K. Nussenzweig M. C. (1996). Requirement for Ku80 in Growth and Immunoglobulin V(D)J Recombination. Nature 382 (6591), 551–555. 10.1038/382551a0 8700231

[B79] Oettinger M. A. Schatz D. G. Gorka C. Baltimore D. (1990). RAG-1 and RAG-2, Adjacent Genes that Synergistically Activate V(D)J Recombination. Science 248 (4962), 1517–1523. 10.1126/science.2360047 2360047

[B80] Oksenych V. Alt F. W. Kumar V. Schwer B. Wesemann D. R. Hansen E. (2012). Functional Redundancy between Repair Factor XLF and Damage Response Mediator 53BP1 in V(D)J Recombination and DNA Repair. Proc. Natl. Acad. Sci. U S A. 109 (7), 2455–2460. 10.1073/pnas.1121458109 22308489 PMC3289340

[B81] Outters P. Jaeger S. Zaarour N. Ferrier P. (2015). Long-Range Control of V(D)J Recombination & Allelic Exclusion: Modeling Views. Adv. Immunol. 128, 363–413. 10.1016/bs.ai.2015.08.002 26477371

[B82] Ouyang H. Nussenzweig A. Kurimasa A. Soares V. C. Li X. Cordon-Cardo C. (1997). Ku70 Is Required for DNA Repair but Not for T Cell Antigen Receptor Gene Recombination *In Vivo* . J. Exp. Med. 186 (6), 921–929. 10.1084/jem.186.6.921 9294146 PMC2199057

[B83] Pan-Hammarstrom Q. Jones A. M. Lahdesmaki A. Zhou W. Gatti R. A. Hammarstrom L. (2005). Impact of DNA Ligase IV on Nonhomologous End Joining Pathways during Class Switch Recombination in Human Cells. J. Exp. Med. 201 (2), 189–194. 10.1084/jem.20040772 15657289 PMC2212791

[B84] Panchakshari R. A. Zhang X. Kumar V. Du Z. Wei P. C. Kao J. (2018). DNA Double-Strand Break Response Factors Influence End-Joining Features of IgH Class Switch and General Translocation Junctions. Proc. Natl. Acad. Sci. U S A. 115 (4), 762–767. 10.1073/pnas.1719988115 29311308 PMC5789958

[B85] Perkins E. J. Nair A. Cowley D. O. Van Dyke T. Chang Y. Ramsden D. A. (2002). Sensing of Intermediates in V(D)J Recombination by ATM. Genes Dev. 16 (2), 159–164. 10.1101/gad.956902 11799059 PMC155324

[B86] Peron S. Laffleur B. Denis-Lagache N. Cook-Moreau J. Tinguely A. Delpy L. (2012). AID-driven Deletion Causes Immunoglobulin Heavy Chain Locus Suicide Recombination in B Cells. Science 336 (6083), 931–934. 10.1126/science.1218692 22539552

[B87] Pilzecker B. Jacobs H. (2019). Mutating for Good: DNA Damage Responses during Somatic Hypermutation. Front. Immunol. 10, 438. 10.3389/fimmu.2019.00438 30915081 PMC6423074

[B88] Polo J. M. Ci W. Licht J. D. Melnick A. (2008). Reversible Disruption of BCL6 Repression Complexes by CD40 Signaling in normal and Malignant B Cells. Blood 112 (3), 644–651. 10.1182/blood-2008-01-131813 18487509 PMC2481532

[B89] Ranuncolo S. M. Polo J. M. Dierov J. Singer M. Kuo T. Greally J. (2007). Bcl-6 Mediates the Germinal center B Cell Phenotype and Lymphomagenesis through Transcriptional Repression of the DNA-Damage Sensor ATR. Nat. Immunol. 8 (7), 705–714. 10.1038/ni1478 17558410

[B90] Raval P. Kriatchko A. N. Kumar S. Swanson P. C. (2008). Evidence for Ku70/Ku80 Association with Full-Length RAG1. Nucleic Acids Res. 36 (6), 2060–2072. 10.1093/nar/gkn049 18281312 PMC2330247

[B91] Reina-San-Martin B. Chen H. T. Nussenzweig A. Nussenzweig M. C. (2004). ATM Is Required for Efficient Recombination between Immunoglobulin Switch Regions. J. Exp. Med. 200 (9), 1103–1110. 10.1084/jem.20041162 15520243 PMC2211855

[B92] Reina-San-Martin B. Chen J. Nussenzweig A. Nussenzweig M. C. (2007). Enhanced Intra-switch Region Recombination during Immunoglobulin Class Switch Recombination in 53BP1-/- B Cells. Eur. J. Immunol. 37 (1), 235–239. 10.1002/eji.200636789 17183606

[B93] Reina-San-Martin B. Difilippantonio S. Hanitsch L. Masilamani R. F. Nussenzweig A. Nussenzweig M. C. (2003). H2AX Is Required for Recombination between Immunoglobulin Switch Regions but Not for Intra-switch Region Recombination or Somatic Hypermutation. J. Exp. Med. 197 (12), 1767–1778. 10.1084/jem.20030569 12810694 PMC2193951

[B94] Roco J. A. Mesin L. Binder S. C. Nefzger C. Gonzalez-Figueroa P. Canete P. F. (2019). Class-Switch Recombination Occurs Infrequently in Germinal Centers. Immunity 51 (2), 337–350. e337. 10.1016/j.immuni.2019.07.001 31375460 PMC6914312

[B95] Rogier M. Moritz J. Robert I. Lescale C. Heyer V. Abello A. (2021). Fam72a Enforces Error-Prone DNA Repair during Antibody Diversification. Nature 600 (7888), 329–333. 10.1038/s41586-021-04093-y 34819671

[B96] Rogozin I. B. Diaz M. (2004). Cutting Edge: DGYW/WRCH Is a Better Predictor of Mutability at G:C Bases in Ig Hypermutation Than the Widely Accepted RGYW/WRCY Motif and Probably Reflects a Two-step Activation-Induced Cytidine Deaminase-Triggered Process. J. Immunol. 172 (6), 3382–3384. 10.4049/jimmunol.172.6.3382 15004135

[B97] Ru H. Chambers M. G. Fu T. M. Tong A. B. Liao M. Wu H. (2015). Molecular Mechanism of V(D)J Recombination from Synaptic RAG1-RAG2 Complex Structures. Cell 163 (5), 1138–1152. 10.1016/j.cell.2015.10.055 26548953 PMC4690471

[B98] Ru H. Mi W. Zhang P. Alt F. W. Schatz D. G. Liao M. (2018). DNA Melting Initiates the RAG Catalytic Pathway. Nat. Struct. Mol. Biol. 25 (8), 732–742. 10.1038/s41594-018-0098-5 30061602 PMC6080600

[B99] Schatz D. G. Oettinger M. A. Baltimore D. (1989). The V(D)J Recombination Activating Gene, RAG-1. Cell 59 (6), 1035–1048. 10.1016/0092-8674(89)90760-5 2598259

[B100] Strom L. Lindroos H. B. Shirahige K. Sjogren C. (2004). Postreplicative Recruitment of Cohesin to Double-Strand Breaks Is Required for DNA Repair. Mol. Cel 16 (6), 1003–1015. 10.1016/j.molcel.2004.11.026 15610742

[B101] Sun X. Bai J. Xu J. Xi X. Gu M. Zhu C. (2021). Multiple DSB Resection Activities Redundantly Promote Alternative End Joining-Mediated Class Switch Recombination. Front Cel Dev Biol 9, 767624. 10.3389/fcell.2021.767624 PMC8671047 34926456

[B102] Teng G. Maman Y. Resch W. Kim M. Yamane A. Qian J. (2015). RAG Represents a Widespread Threat to the Lymphocyte Genome. Cell 162 (4), 751–765. 10.1016/j.cell.2015.07.009 26234156 PMC4537821

[B103] Teng G. Schatz D. G. (2015). Regulation and Evolution of the RAG Recombinase. Adv. Immunol. 128, 1–39. 10.1016/bs.ai.2015.07.002 26477364

[B104] van Gent D. C. McBlane J. F. Ramsden D. A. Sadofsky M. J. Hesse J. E. Gellert M. (1995). Initiation of V(D)J Recombination in a Cell-free System. Cell 81 (6), 925–934. 10.1016/0092-8674(95)90012-8 7781069

[B105] Wei H. Yu X. (2016). Functions of PARylation in DNA Damage Repair Pathways. Genomics Proteomics Bioinformatics 14 (3), 131–139. 10.1016/j.gpb.2016.05.001 27240471 PMC4936651

[B106] Weitering T. J. Takada S. Weemaes C. M. R. van Schouwenburg P. A. van der Burg M. (2021). ATM: Translating the DNA Damage Response to Adaptive Immunity. Trends Immunol. 42 (4), 350–365. 10.1016/j.it.2021.02.001 33663955

[B107] Xu G. Chapman J. R. Brandsma I. Yuan J. Mistrik M. Bouwman P. (2015). REV7 Counteracts DNA Double-Strand Break Resection and Affects PARP Inhibition. Nature 521 (7553), 541–544. 10.1038/nature14328 25799992 PMC4671316

[B108] Yan C. T. Boboila C. Souza E. K. Franco S. Hickernell T. R. Murphy M. (2007). IgH Class Switching and Translocations Use a Robust Non-classical End-Joining Pathway. Nature 449 (7161), 478–482. 10.1038/nature06020 17713479

[B109] Yeap L. S. Meng F. L. (2019). Cis- and Trans-factors Affecting AID Targeting and Mutagenic Outcomes in Antibody Diversification. Adv. Immunol. 141, 51–103. 10.1016/bs.ai.2019.01.002 30904133

[B110] Yin B. Savic V. Juntilla M. M. Bredemeyer A. L. Yang-Iott K. S. Helmink B. A. (2009). Histone H2AX Stabilizes Broken DNA Strands to Suppress Chromosome Breaks and Translocations during V(D)J Recombination. J. Exp. Med. 206 (12), 2625–2639. 10.1084/jem.20091320 19887394 PMC2806628

[B111] Zha S. Guo C. Boboila C. Oksenych V. Cheng H. L. Zhang Y. (2011a). ATM Damage Response and XLF Repair Factor Are Functionally Redundant in Joining DNA Breaks. Nature 469 (7329), 250–254. 10.1038/nature09604 21160472 PMC3058373

[B112] Zha S. Jiang W. Fujiwara Y. Patel H. Goff P. H. Brush J. W. (2011b). Ataxia Telangiectasia-Mutated Protein and DNA-dependent Protein Kinase Have Complementary V(D)J Recombination Functions. Proc. Natl. Acad. Sci. U S A. 108 (5), 2028–2033. 10.1073/pnas.1019293108 21245310 PMC3033273

[B113] Zhang X. Yoon H. S. Chapdelaine-Williams A. M. Kyritsis N. Alt F. W. (2021). Physiological Role of the 3'IgH CBEs Super-anchor in Antibody Class Switching. Proc. Natl. Acad. Sci. U S A. 118 (3). 10.1073/pnas.2024392118 PMC7826415 33441485

[B114] Zhang X. Zhang Y. Ba Z. Kyritsis N. Casellas R. Alt F. W. (2019a). Fundamental Roles of Chromatin Loop Extrusion in Antibody Class Switching. Nature 575 (7782), 385–389. 10.1038/s41586-019-1723-0 31666703 PMC6856444

[B115] Zhang Y. Zhang X. Ba Z. Liang Z. Dring E. W. Hu H. (2019b). The Fundamental Role of Chromatin Loop Extrusion in Physiological V(D)J Recombination. Nature 573 (7775), 600–604. 10.1038/s41586-019-1547-y 31511698 PMC6867615

[B116] Zhang Y. Zhang X. Dai H. Q. Hu H. Alt F. W. (2022). The Role of Chromatin Loop Extrusion in Antibody Diversification. Nat. Rev. Immunol. 1, 1. 10.1038/s41577-022-00679-3 PMC9376198 35169260

[B117] Zhao B. Rothenberg E. Ramsden D. A. Lieber M. R. (2020). The Molecular Basis and Disease Relevance of Non-homologous DNA End Joining. Nat. Rev. Mol. Cel Biol 21 (12), 765–781. 10.1038/s41580-020-00297-8 PMC8063501 33077885

[B118] Zhu C. Bogue M. A. Lim D. S. Hasty P. Roth D. B. (1996). Ku86-deficient Mice Exhibit Severe Combined Immunodeficiency and Defective Processing of V(D)J Recombination Intermediates. Cell 86 (3), 379–389. 10.1016/s0092-8674(00)80111-7 8756720

